# Spatial transcriptome analysis of the human eyelid depicts meibomian gland cell differentiation: A pilot study

**DOI:** 10.14814/phy2.70571

**Published:** 2025-09-19

**Authors:** Hans Binder, Ulrike Hampel, Henry Loeffler‐Wirth, Florian Hansmann, Helga Pfannkuche, Maria Schmidt, Marlon R. Schneider

**Affiliations:** ^1^ Interdisciplinary Institute for Bioinformatics (IZBI) University of Leipzig Leipzig Germany; ^2^ Armenian Bioinformatics Institute and Institute for Molecular Biology Yerevan Armenia; ^3^ Department of Ophthalmology University Hospital Leipzig Leipzig Germany; ^4^ Institute for Veterinary Pathology University of Leipzig Leipzig Germany; ^5^ Institute of Veterinary Physiology University of Leipzig Leipzig Germany

**Keywords:** eye, Meibomian gland, pseudotime, sebaceous gland, skin, spatial transcriptomics

## Abstract

Meibomian glands (MGs) are an integral component of the ocular defense system, as their secretion product, meibum, is essential for protecting the eye surface. To characterize the transcriptional program underlying meibum production, we employed spatial transcriptomics (ST) analysis of the human eyelid from a sample from a 60‐year‐old male. We resolved 18 distinct eyelid clusters, representing structures such as the conjunctiva, epidermis, hair‐associated sebaceous glands, and MGs. Focusing on the MG, we distinguished basal (MEI‐B cluster) and differentiating (MEI‐DIFF cluster) meibocytes, as well as a third, duct‐related cluster (MEI‐DUCT). Self‐organizing maps (SOM) portrayal of ST images and pseudotime analysis confirmed progress from MEI‐B to MEI‐DIFF and further to MEI‐DUCT, as the latter turned out to include terminally differentiated meibocytes. Accordingly, gene set enrichment analysis associated early/intermediate meibocyte maturation with energy and lipid metabolism, and later stages with barrier functions. We also identified significant differences between the MG and sebaceous gland transcriptomes. The MG‐specific signature included transcripts such as *AQP9*, *MMP3*, and *PITX1*, and selective expression of PITX1 in the MG compared to the sebaceous gland was confirmed by immunohistochemistry on the same sample and samples from three other elderly adults. We deliver the first spatial portrait of the human MG transcriptional landscape. Besides enhancing our understanding of MG physiology, our study identifies novel targets for regulating MG homeostasis in health and disease.

## Graphical abstract

Spatial transcriptome analysis of the human Meibomian gland.

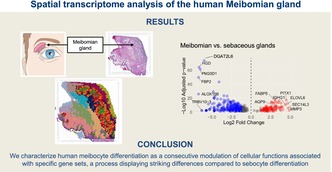

## 1 INTRODUCTION

The eye surface is covered by a thin fluid film that serves several critical functions. It lubricates the eye, delivers nutrients and growth factors to the epithelium, and serves as a barrier to the outside environment (de Souza et al., 2006; Dilly, 1994). The tear film is usually stratified into three layers: an internal mucus layer, a middle aqueous layer, and an outer lipid layer at the eye‐air interface (Willcox et al., 2017). The lipid layer, a complex mixture of lipids of different classes, avoids tear evaporation and is argued to allow the spread of the tear film and to prevent its collapse onto the ocular surface (Butovich, 2013; Millar & Schuett, 2015).

The main source of tear lipids is meibomian glands (MGs), specialized sebaceous glands embedded within the tarsal plates of the eyelids that continuously produce and excrete meibum, a lipid‐rich material via a ductal system directly onto the ocular surface (Butovich, 2017; Knop et al., 2011). The MG acini follow a holocrine secretion mode, as peripheral cells progressively synthesize lipids and are dislodged to the central part of the gland, where the mature meibocytes rupture and are released together with their oily content via a central duct at the orifice located at the edge of the eyelids. The quantity and chemical composition of meibum produced by MGs are crucial for maintaining ocular health, and a number of ocular pathologies, including meibomian gland dysfunction (MGD), with an impressive prevalence of 40% and dry eye disease (especially its evaporative form), have been linked to either insufficient meibum production or delivery, or an abnormal lipid composition (Franck, 1991; Yazdani et al., 2019).

Meibum lipids differ considerably from those of other tissues and secretions, including those found in skin sebaceous gland‐derived sebum (Butovich & Suzuki, 2020; Liu et al., 2024). It is therefore likely that the molecular pathways underlying meibogenesis are different from those in other organs and tissues. Previous studies, frequently founded on the phenotype of genetically modified mice (Ehrmann & Schneider, 2016), revealed a number of enzymes that are involved in the biosynthesis of meibum, including wax ester synthases (AWATs) (Sawai et al., 2021; Widjaja‐Adhi et al., 2020), very long‐chain fatty acid elongases (ELOVLs) (Butovich et al., 2019; McMahon et al., 2014), fatty acid desaturases (Miyazaki et al., 2001), cholesteryl ester synthases (SOATs) (Butovich et al., 2021), and numerous other gene products that bring about the various steps of meibogenesis (Butovich, 2017). Furthermore, untargeted transcriptome analyses based on microarrays or RNA sequencing revealed gene expression differences in MG between females and males (Butovich et al., 2019), as well as MG transcriptional changes during tumorigenesis (Kumar et al., 2007), aging (Parfitt et al., 2016), or after feeding a high‐fat diet (Zou et al., 2023). More recently, single‐cell RNA sequencing (scRNAseq) of mouse tarsal plates (Butovich & Wilkerson, 2022; Wiedemann et al., 2024) provided novel insights into the gene expression signatures of MG and surrounding cellular components. Although mouse meibum has a chemical composition more similar to human meibum than that of dog and rabbit meibum (Butovich et al., 2012), differences exist. Furthermore, the members of important enzyme families related to lipid metabolism differ between mice and humans. As an example, the gene encoding Scd3, a member of the stearoyl‐coenzyme A desaturase (SCD) family, strongly expressed in the mouse MG (Dahlhoff et al., 2016; Zheng et al., 2001), is absent in the human genome. Thus, while mouse‐based studies provide valuable information on meibocyte differentiation and the underlying transcriptional program, our understanding of the human meibocyte transcriptional landscape remains fragmentary.

While single‐cell transcriptomics is a powerful tool to segregate cell types according to their gene expression program, it does not directly provide information on tissue organization and cell–cell interactions. Spatial transcriptomics (ST), in contrast, unifies transcriptional profiles and spatial information within the native tissue context (Rao et al., 2021). In the present study, we assessed the human eyelid based on a sample from a 60‐year‐old male with untargeted spatial transcriptomics to reveal its cellular composition and, in particular, to assess the pathways governing MG homeostasis and differentiation. In addition, the availability of ST data for the human sebaceous gland (Schmidt et al., 2024) and the presence of hair follicle‐associated sebaceous glands in the analyzed eyelid sample allowed a direct comparison between the sebaceous and meibomian transcriptome.

## 2 METHODS

### 2.1 Tissue sample processing

The collection and use of the eyelid samples was approved by the ethics committee of the medical faculty of the University of Leipzig (070‐22‐ek). The eyelid samples employed in this study were collected at the Department of Ophthalmology of the University of Leipzig during lateral tarsal strip procedure for ectropion correction and after patient consent for use of the specimen for research purposes, and in full compliance with the Declaration of Helsinki principles. The patients were a 60 years old man (patient 1) and three women aged 85 (patient 2), 99 (patient 3), and 70 years (patient 4). Such samples typically exhibit redness/inflammation due to the irritation of the conjunctiva. Patient 1 was employed for the spatial transcriptome analysis; all four patients were employed for immunofluorescence staining. The samples were formalin‐fixed, paraffin‐embedded, and spatial transcriptomic analysis was carried out for a single run with patient 1's sample using the Visium platform (10× Genomics) according to the manufacturer's instructions by a qualified service provider (Indivumed Services, Hamburg, Germany). Sequencing data were processed using the Space Ranger software (10× Genomics). Quality control metrics, including library saturation and feature‐spot distribution assessment, were evaluated and found within the recommended range.

### 2.2 Bioinformatic analysis

Sequencing data were aligned to the human genome (10× Genomics Visium Space Ranger software v.2.0.0 with GRCh38) to quantify gene expression for downstream analyses. All analytical workflows were performed in R (v4.4.0). Processed datasets are available upon request.

The analysis pipeline started with dimensionality reduction and visualization using the Seurat R package (version 5.0.0; Hao et al., 2024). Preprocessing steps involved filtering low‐quality spots and normalizing gene expression levels. Principal component analysis (PCA) was applied to identify sources of variance and uncover the underlying data structure. Uniform Manifold Approximation and Projection (UMAP) was used with default parameters to visualize high‐dimensional data in two dimensions.

Spots were clustered using the shared nearest neighbor (SNN) modularity optimization algorithm based on gene expression profiles. Differentially expressed genes (DEGs) between clusters were identified using the Seurat FindMarkers function with standard settings, employing the Wilcoxon Rank Sum test and a logfc.threshold of 0.1. Each cluster was assigned to a functional skin cell type, and annotations were linked with Hematoxylin and Eosin (H&E) stained images and UMAP plots. This allowed the integration of spatial and transcriptomic data to reveal the molecular characteristics and spatial distributions of skin cell types (Table S1). To explore meibocyte differentiation dynamics, we applied Monocle 3 (version 1.0.0; (Cao et al., 2019)). A principal graph was fitted to the meibocyte partition in UMAP space, with cluster MEI‐B defined as the starting point of differentiation. Spots were ordered along the pseudotime trajectory, and DEGs across pseudotime were identified to uncover dynamic changes during meibocyte maturation.

Functional enrichment analysis of these DEGs was performed using DAVID Bioinformatics Resources to reveal associated biological processes, pathways, and functional categories. To provide spatial context, the average expression of metagene modules derived from enrichment analysis was mapped onto tissue images, integrating functional and transcriptomic data.

High‐dimensional expression data of 15,801 gene transcripts was reduced to 3902 metagenes using the oposSOM software (version 2.2.8; (Löffler‐Wirth et al., 2015)). These metagenes were organized into a 55 × 55 grid, grouping genes with similar expression profiles. Individual spot transcriptomes were visualized as “SOM portraits,” with red indicating overexpressed and blue indicating underexpressed metagenes in a maximum‐to‐minimum expression scale. The self‐organizing properties of the SOM highlighted clusters of correlated genes, with modules detected based on the overexpression criterion described previously (Wirth et al., 2011).

For comparative analysis, we examined 500 DEGs identified from the pseudotemporal (PT) analysis of sebaceous gland hyperplasia (SGH) reported by Schmidt et al. (2024) alongside 500 DEGs derived from PT analysis of meibocyte maturation. We visualized shared and unique genes using volcano plots, Venn diagrams, and Sankey flow diagrams to highlight overlapping molecular signatures and distinct features.

We further conducted DEG analyses between MEI and EL‐SEB (MEI = meibocytes, EL = eyelid, SEB = sebocytes) and between MEI and SGH‐SEB to identify transcriptional differences underlying meibum and sebum secretion. Data sets were integrated using Seurat's standard integration pipeline to generate an integrated embedding, followed by the identification of differential markers on the spatial assay with FindMarkers (Wilcoxon Rank Sum test, logfc.threshold = 0.1). Gene set enrichment analysis was performed on these DEGs to reveal functional associations, providing deeper insights into the biological processes specific to MEI and EL‐SEB.

### 2.3 Immunofluorescence

For the detection of DGAT2L6 and PITX1, 5 μm‐thick tissue was deparaffinized and cooked for 10 min in 0.01 M citrate buffer (pH 6.0) for antigen demasking. They were next washed (3 × 3 min) in Tris‐buffered saline (TBS), preincubated for 60 min in TBS containing 5% bovine serum albumin, and incubated overnight with primary rabbit‐anti‐DGAT2L6 antibodies (dilution 1:200; ABIN6275058; antibodies‐online, Aachen, Germany) or with rabbit‐anti‐PITX1 antibodies (dilution 1:100; 10873‐1‐AP, Proteintech, Planegg‐Martinsried, Germany). After incubation with the primary antibodies, the specimens were washed three times in TBS and incubated for 2.5 h at room temperature in buffer solution containing secondary anti‐rabbit antibodies raised in goats conjugated to indocarbocyanine (Cy3, SA00009‐2, Proteintech, Planegg‐Martinsried, Germany) in a dilution of 1:500. To stain nuclei, sections were incubated for 1 min at room temperature with 4′,6‐diamidino‐2‐phenylindole (DAPI, Sigma‐Aldrich, Taufkirchen, Germany) in a dilution of 1:4000. Finally, the specimens were washed in TBS, coverslipped with Prolong Gold Antifade Mountant (P10144 Invitrogen, Darmstadt, Germany), and stored at 4°C.

The stained sections were analyzed using a fluorescence microscope (Zeiss Axio Observer 7 Apotome, Carl Zeiss, Jena, Germany). Employed objectives were EC Plan‐Neofluar 10×/0.3 and Plan‐Apochromat 20×/0.8 (both from Carl Zeiss, Jena, Germany). Photomicrographs were taken with a black and white camera (Axiocam 705 mono R2, Carl Zeiss, Jena, Germany) attached to an image analysis system (ZEN 3.6 (blue edition), Carl Zeiss, Jena, Germany).

## 3 RESULTS

### 3.1 Unsupervised clustering distinguishes 18 cell states in the human eyelid

**FIGURE 1:**
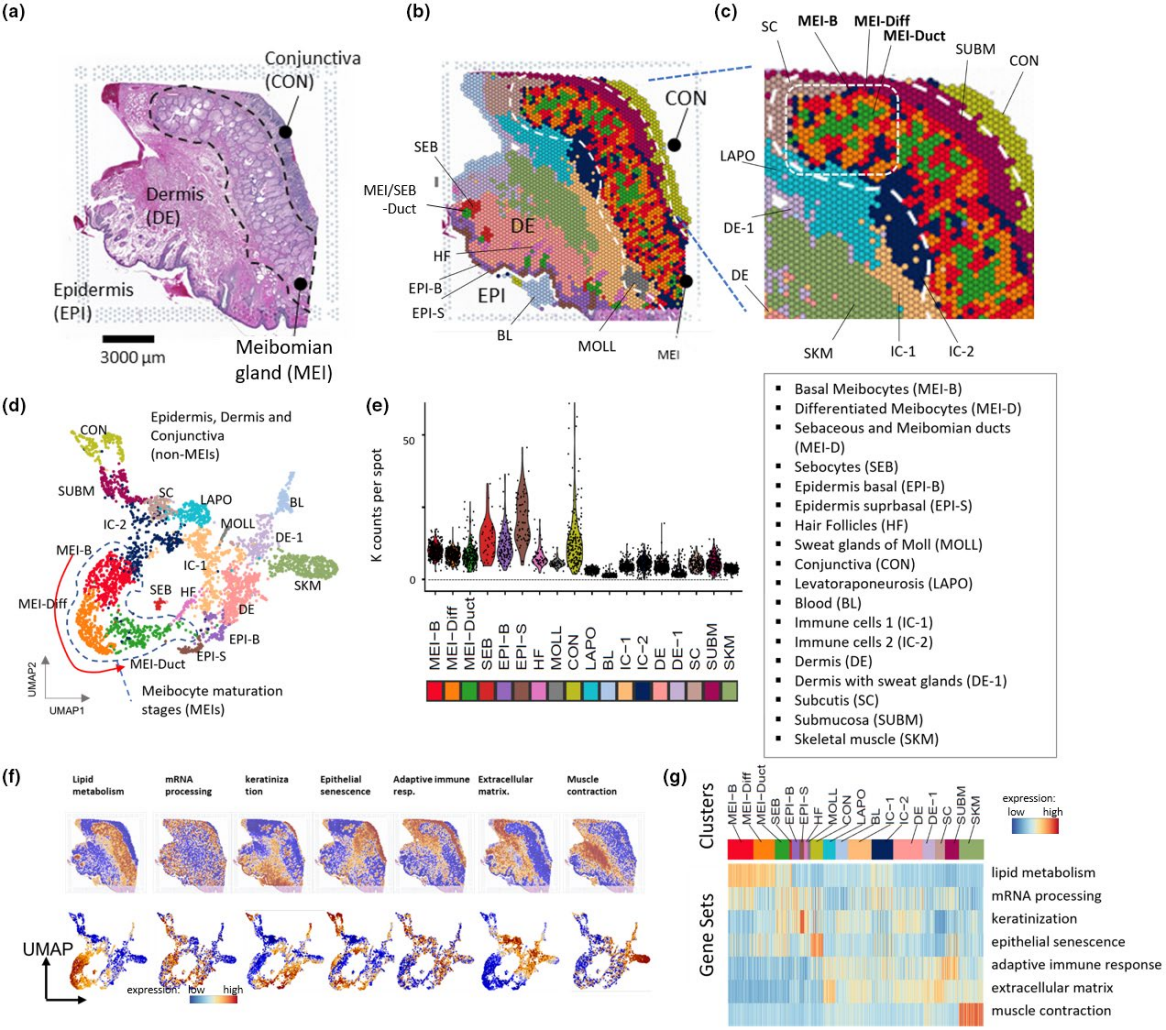
Overview of the eyelid ST analysis. (a) H&E‐stained and (b) spatial transcriptomics images of the eyelid. Colors of the spots refer to different cell types (see legend in the figure). (c) Enlargement of the ST image in the region of Meibomian glands. (d) UMAP of the spots distributes cell type‐specific clusters along two major groups, one representing the putative maturation of meibocytes from MEI‐B to MEI‐DIFF (and, with the restrictions discussed in the main text, to MEI‐DUCT) and the other one the remaining clusters. (e) Average transcript counts for clusters per spot. (f) Expression of selected functional gene sets in the ST images (upper row) and in the UMAP (lower row). (g) A heatmap showing the relatedness between the cell types and the expression of the functional gene sets. Brown and blue in (f) and (g) indicate high and low expression, respectively.

Figure 1a,b compare the microscopic images of the human eyelid using H&E staining and ST‐generated clusters, respectively. In total, 18 cell states were obtained by unsupervised clustering based on gene expression similarities, which, combined with histological assessment, identified three clusters of the meibomian gland (MEI‐B, MEI‐DIFF, and MEI‐DUCT), single clusters referring to sebaceous glands (SEB), hair follicles (HF), sweat glands of moll (MOLL), conjunctiva (CON), submucosa (SUBM), subcutis (SC), blood (BL), levator aponeurosis (LAPO), and skeletal muscle (SKM), two epidermal clusters (EPI‐B and EPI‐S), two immune cell‐enriched clusters (IC‐1 and IC‐2), and two dermal clusters (DE and DE‐1). Top expressed genes of each cluster were provided in Table S1. Figure 1c zooms into the meibomian gland area and shows layered structures which can be assigned to meibocyte differentiation stages, namely basal (MEI‐B) and differentiated meibocytes (MEI‐DIFF) as well as ducts (MEI‐DUCT). Interestingly, the latter cluster (green) is also found near sebocytes (SEB) and hair follicles (HF), thus representing the duct of both meibomian and sebaceous glands.

A Uniform Manifold Approximation and Projection (UMAP) plot (Figure 1d) was employed to cluster the ST‐spots according to the similarities of their transcriptomes without considering the spatial context of the microscopic images. One main branch forms along the differentiation trajectory of the different meibocyte types (MEI‐B, MEI‐DIFF, and MEI‐DUCT, marked with the dashed line and the red arrow), with other spots clustering nearby. The highest transcript counts per spot were detected for EPI‐S, EPI‐B, SEB, and CON as well as for the MG‐related spots, while the BL spot shows, as expected, the lowest transcript counts (Figure 1e). Coloring of the ST images and the UMAP utilizing selected gene sets reveals the functional context of different spatial areas and cell types (Figure 1f). The gene set “lipid metabolism” colors the MG region in the ST image and the MG‐related spots in the UMAP in brown, while “keratinization” marks the EPI‐regions, “epithelial senescence” the conjunctiva, “adaptive immune response” the submucosa, “extracellular matrix” the LAPO, and “muscle contraction” the SKM regions and spots (Figure 1f). A heatmap provides an overview of the relatedness between the cell types and the expression of the functional gene sets (Figure 1g).

### 3.2 Resolving and interpreting the spatial transcriptomic landscapes

**FIGURE 2:**
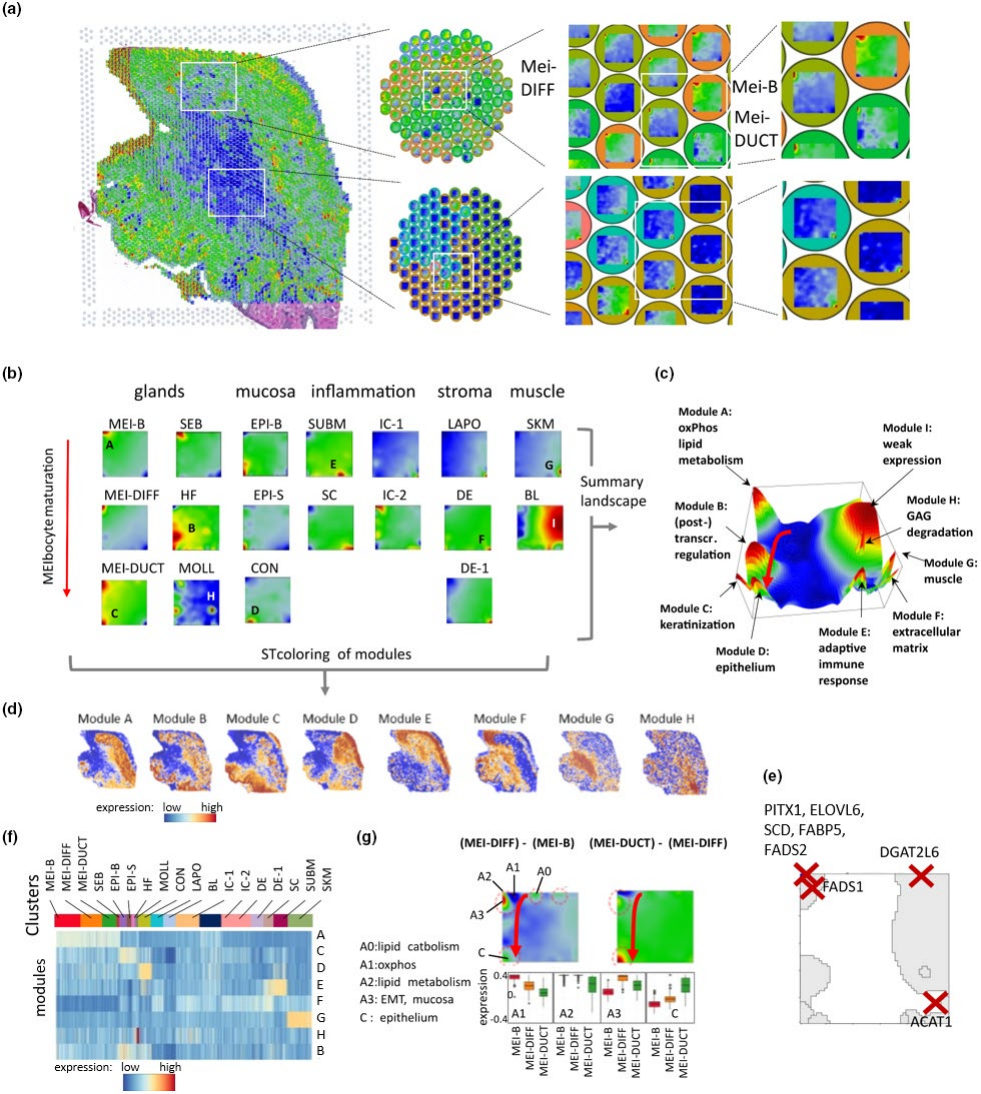
SOM portrayal of the eyelid ST. (a) Each spot of the ST image is characterized by its SOM expression portrait. The consecutive zoom‐in steps illustrate their diversity and spatial arrangement. (b) Mean portraits averaged over all spots of the same cluster (see Figure 1b) show characteristic patterns as red and blue gene modules over‐ and under‐expressed in the respective cluster. (c) The summary landscape collects all identified modules together. Their functional context was obtained using gene set enrichment analysis. (d) The ST images were colored according to the expression of the different modules (compare with Figure 1f). (e) Map of selected key genes related to meibocyte maturation located in spot A (compare with c). (f) The heatmap shows the expression of modules across the cell types. (g) Difference portraits between the meibocyte maturation stages reveal details of specific transcriptional programs in module A marked as A0 to A3 and C. Their expression levels are shown as a boxplot.

To better assess the cellular heterogeneity of the sample, we next applied self‐organizing maps (SOM) portrayal of ST images (Schmidt et al., 2024). This machine learning‐based method generates a transcriptomic “portrait” for each of the 3902 spots of the ST image of the eyelid, which enables their mutual comparison and analysis by visual inspection. The consecutive zoom‐in steps illustrate their individual diversity and spatial arrangement related to the cell composition of the spots and their spot cluster assignments (Figure 2a). Over‐ and under‐expressed modules of co‐regulated genes are colored in red and blue, respectively, giving rise to a specific blurry red‐to‐blue texture of each of the spot portraits and, in turn, of the whole ST image (Figure 2a, see also Figure S1 for an overview of the SOM analysis). Mean portraits of the spot clusters, calculated by averaging over all respective spot portraits, revealed specific expression patterns of (red‐colored) modules of upregulated genes (Figure 2b).

Gene set enrichment analysis of the genes in each of the modules reveals their functional context, establishing a transcriptomic overview landscape of the eyelid (Figure 2c and Figure S1a). It consists of “mountains” of genes specifically overexpressed in certain cell types which associate with functional themes as lipid metabolism (module A; MEI‐B, MEI‐DIFF, SEB), posttranslational modification and protein degradation (module B; HF and MOLL), keratinization and mucosa (module C; MEI‐DUCT, EPI‐B and EPI‐S), epithelium (module D; CON), inflammation and immune response (module E; SUBM), extracellular matrix (module F; IC‐1, LAPO, DE), skeletal muscle (module G; SKM), and glucosaminoglycan degradation (module H; MOLL). Table S2 includes the genes in each of these modules. As module I contains a variety of weakly expressed and low variant genes (see low transcript count of BL in Figure 1e, weak expression of module I in Figure 2g, and low transcript variance in spot I in Figure S1b) that were assigned as blood‐related, we exclude it from further analysis to reduce background noise. We colored the ST images using the expression of the identified modules A–H, which marks the respective areas in brown (Figure 2d); for example, module A is indicative for the area of the MG (and SEB) and module E for SUBM‐areas, which confirms the association between the module expression and the histological assignment in the ST image (Figure 2d, compare with Figure 1f). Location of selected genes in the SOM associates with their function; for example, genes related to meibogenesis such as *ELOVL6*, *SCD*, *FABP5*, and *FADS2* locate in module A (Figure 2e). The expression level of the modules across the cell types indicates specific, spot cluster‐related upregulation of most of the modules; for example, of module C (keratinization) for the MOLL cluster, while a few clusters show moderate upregulation across several spot clusters; for example, module A (oxphos and lipid metabolism) across the MEI clusters (Figure 2f).

Notably, the SOM provides a topology‐aware gene expression landscape of the eyelid, which locates similar modules nearby (e.g., modules related to meibogenesis) and anticorrelated ones at distant positions (e.g., modules related to meibogenesis and skeletal muscle; Figure 2c and Figure S1d). Importantly, part of the modules co‐express in the mean portraits of different tissues (e.g., module A and C in MEI‐DUCT; A and F in IC‐2, C and E in SUBM), which reflects overlap of different cell types (and functions) captured in the respective spots, for example, of mature meibocytes and of ductal cells in MEI‐DUCT (see Figure 2b, Figure S1D–G, and next subsection). The ST data of the eyelid presented here can be interactively explored in the ST‐browser (Schmidt, Avagyan, et al., 2024) under the link provided in the data availability statement below.

Taken together, SOM‐portrayal provides a comprehensive description of the expression landscape of the eyelid, which deciphers its diversity as well as overlapping functions and possible trajectories of meibocyte differentiation.

### 3.3 Spatial transcriptomics distinguishes meibocyte maturation stages

MG‐related spots divide into three types referring to basal (MEI‐B), differentiated (MEI‐DIFF), and ductal cells (MEI‐DUCT). Fully mature sebocytes underwenting holocrine secretion have probably not been included in our analysis due to their low or absent transcriptional activity. For a detailed view, we calculated difference SOM portraits between sequential pairwise combinations of the three mean portraits (Figure 2f). The difference between the differentiated and basal meibocytes reveals sub‐modularization of the module A, which indicates the shift of gene activity from energy metabolism (submodule A1) towards lipid metabolism (A2) in the SOM, meaning that the expression of MEI‐DIFF increases relative to MEI‐B in A2 compared with A1. Another difference, this time between MEI‐DIFF and MEI‐DUCT, indicates emerging transcript numbers with epidermal characteristics, representing ductal cells captured together with MEI‐DIFF cells due to the limited resolution of Visium (modules A2 and C). The expression of the submodules shows the shift of maximum expression from MEI‐B to MEI‐DUCT from A1 to C, respectively (Figure 2f). Hence, the three clusters of meibocytes describe the underlying energy and lipid metabolism of meibum production, including intermixing with ductal cells in the late stage. In the UMAP and the SOM, we can draw meibocyte maturation trajectories in spot and gene space (see red arrows in Figures 1d and Figure 2c,g, respectively) pointing from MEI‐B to MEI‐DIFF (and also to the mixed cluster MEI‐DUCT) and the respective upregulated genes and associated transcriptional programs, respectively. Interestingly, the MEI‐DUCT cluster also includes spots referring to the ductal compartment of the sebaceous gland in the eyelid (Figure 1b and below), indicating a similar transcriptional signature. In agreement with previous studies on mouse Meibomian glands (Parfitt et al., 2013; Wiedemann et al., 2024; Yang et al., 2023, 2024), MEI‐DUCT was characterized by high KRT6B and rather low/moderate KRT1/KRT10 transcript levels (Table S1).

### 3.4 Pseudotime analyses detail meibocyte differentiation

**FIGURE 3:**
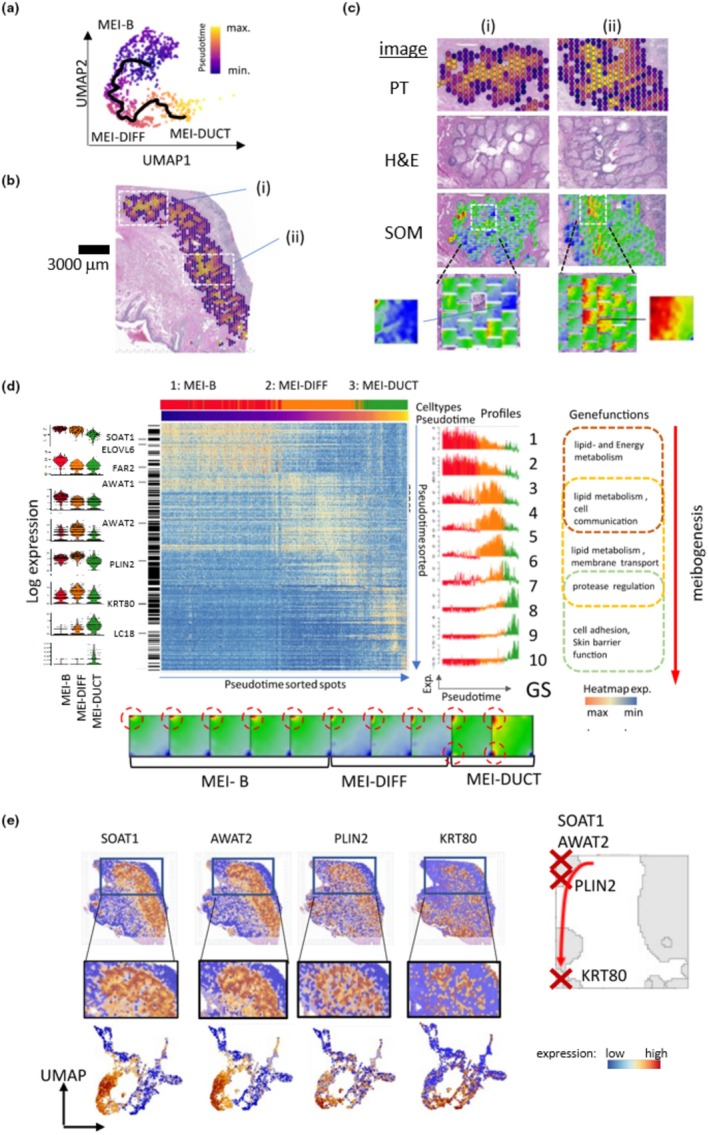
Spatiotemporal dynamics of meibocyte maturation. (a) Pseudotime trajectory across the meibocyte maturation stages in the UMAP plot assigns PT‐values to each spot. (b) PT coloring of the ST image visualizes the maturation dynamics. (c) Zoom‐in areas (i) and (ii) enlarge the microanatomy of MG in PT and H&E coloring. The SOM portraits of the respective spots indicate changes in the transcriptional programs. (d) Expression heatmap of the top 500 DEGs along the PT trajectory. Expression of selected key genes is shown by violin plots across the MEI‐states. Expression profiles of gene sets along the PT (GS1–10) reveal the progressive right shift of maximum expression with increasing PT (right part) as well as changing gene functions with meibocyte differentiation, which associates with consecutive changes in the mean SOM portraits averaged over the respective gene groups (part below). (e) ST images are colored according to the expression level of the key genes. The zoom‐in shows the spatial expression dynamics from the basal layer towards the ductus as a centripetal pattern. Their coloring of the UMAP verifies the shift of overexpression from MEI‐B to MEI‐DUCT areas of the MG branch. The genes locate along the maturation trajectory in the SOM (right part).

For a more detailed view of the meibocyte maturation process, we performed pseudotime (PT) analysis of the MG‐spots in the UMAP by defining its starting point in the MEI‐B cluster (Figure 3a). The PT trajectory crosses through the spots from early (dark blue) to late (yellow, mainly MEI‐DUCT) PT values, which were used to color also the ST image (Figure 3b). The obtained PT image resolves fine structures of the MG, showing centripetal purple‐to‐yellow layered structures visualizing progressive meibocyte maturation (PT coloring in Figure 3b,c). Two zoom‐in regions indicate associations with the H&E image as well as the local spot portraits showing a more blue and red texture for early stage and late stage maturation stages due to activation of module A only and also of modules B and C, respectively (Figure 3c, spot portraits in (i) and (ii), respectively). These data indicate again that the MEI‐DUCT cluster, besides including meibomian and sebaceous duct cells, also includes meibocytes at a more advanced differentiation stage compared to MEI‐DIFF, as evidenced by the co‐expression of spots A and C (Figure 3d).

A heatmap readily sorted differentially expressed genes (DEG) along the PT‐axis, revealing the progressive shift of maximum expression from MEI‐B towards MEI‐DIFF and MEI‐DUCT, with a graded step‐like change between MEI‐B and MEI‐DIFF (Figure 3d, right part, profile of GS 4). We used consecutive windows of 50 genes (GS1‐10) to visualize their collective expression changes as well as selected single gene expression (right and left part of Figure 3d, respectively). Functional annotations using gene set enrichment verify our previous results associating meibocyte maturation with energy and lipid metabolism in early and intermediate PT and with barrier and epithelial functions at late PT. The respective SOM of the GS1‐10 also verifies the shift of upregulated, red gene expression modules in a counterclockwise direction from module A towards module C along the left edge of the portraits (lower part of Figure 3d). Finally, expression‐level coloring of the selected single genes in the ST reproduces the concentric layers found in PT analysis (compare Figure 3b,c with Figure 3e) as well as the PT trajectory in the UMAP (compare lower row in Figure 3e and Figure 3a). Note that these genes locate along the maturation trajectory in the SOM (Figure 3e, right part).

### 3.5 Comparing sebocyte and meibocyte transcriptional programs

**FIGURE 4:**
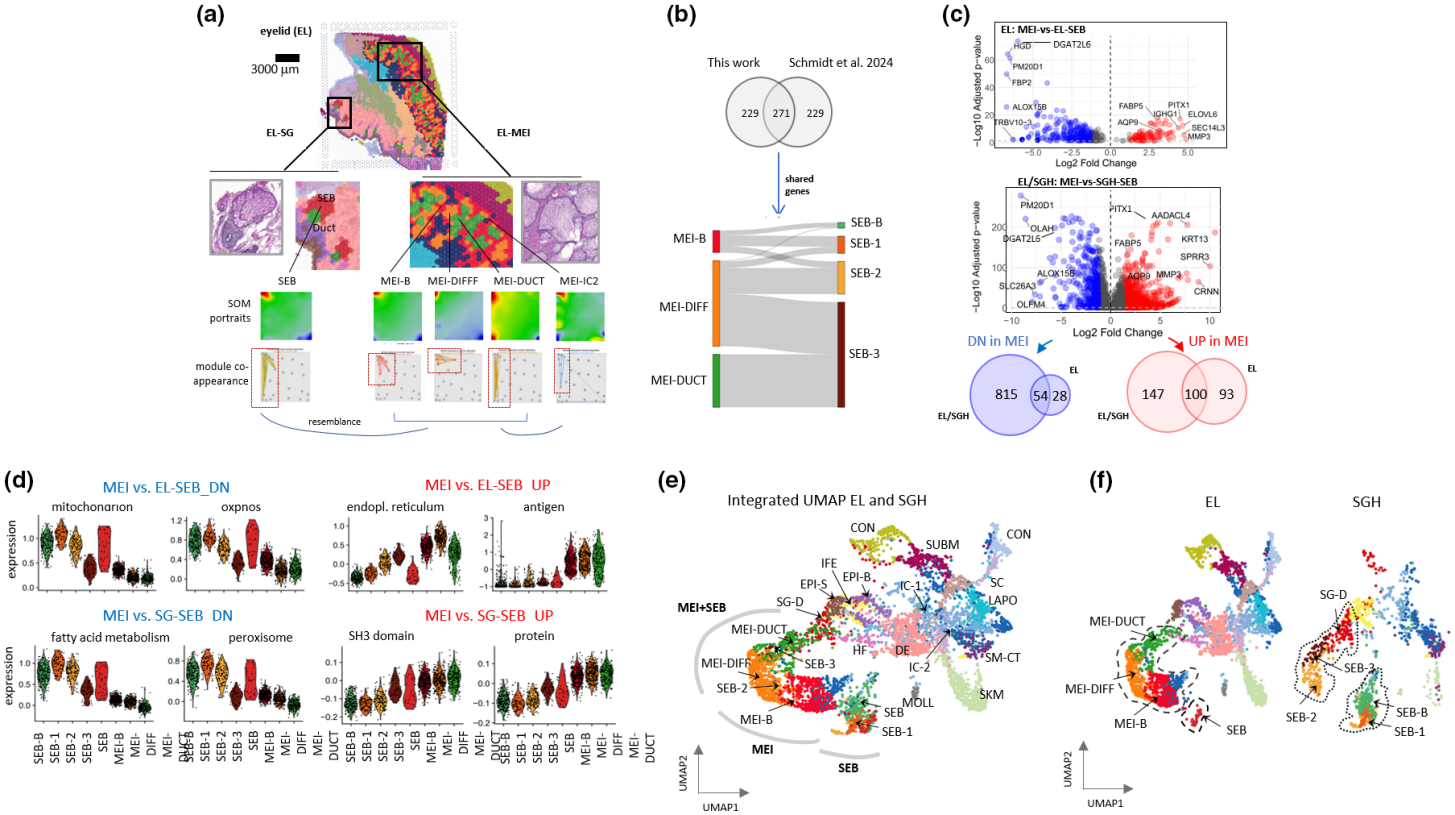
Comparison of meibocyte and sebocyte maturation. (a) The eyelid ST image exhibits EL‐MG and EL‐SG (EL assigns them to the eyelid sample), which share the ductal (MEI‐DUCT) cell types (green) but indicate different cell types SEB (dark red), MEI‐B (red), and MEI‐DIFF (apricot). (b) Comparison of 500 differential pseudotime genes from EL‐MG (this publication) with that of a sebaceous gland hyperplasia (SGH) taken from a previous publication (Schmidt, Hansmann, et al., 2024) indicates 271 shared genes and 229 unique genes on both sides. The shared genes were assigned to different maturation stages of meibocytes and sebocytes, providing a Sankey‐flow diagram between the maturation stages suggesting differences in the transcriptomes of the basal layers in agreement with the ST images (in a). (c) Volcano plots of differential expression analysis between MEI and SG in the eyelid (part above, MEI‐vs‐EL‐SEB) and in the skin (part below, MEI‐vs‐SGH‐SEB). Venn diagrams of up‐and down‐regulated DEGs taken from both Volcano plots show about 50% agreement between EL‐SEB and SGH‐SEB. (d) Violin plots of differentially up‐and down‐expressed gene sets between MEI and SEB in the eyelid and in the skin show the activation or deactivation patterns across the respective maturation stages. (e) Integrated UMAP of the ST spots of the EL (this publication) and SGH (Schmidt, Hansmann, et al., 2024) indicated separate and joint clusters of SEB and MEI related spots, as indicated in the figure. (f) The integrated UMAP was split into spots taken from the EL (left) and the SGH (right). Note the difference between SEB‐B and MEI‐B (basal) spots in both images.

MG are considered specialized sebaceous glands (Schneider & Paus, 2010; Yaba et al., 2024), but the composition of meibum differs considerably from that of sebum, suggesting major differences in the transcriptional program of both glands (Schmidt et al., 2025). The fortuitous presence of hair follicle‐associated sebaceous glands in the analyzed eyelid (EL) sample allowed a direct comparison between the sebaceous and meibomian transcriptomes. The ST analysis revealed the sebaceous gland in the left region of the eyelid image, where sebocytes (SEB) form a distinct cluster (red), while the ductal components of the SG are merged with MEI‐DUCT spots into a single cluster (green, Figure 4a and Figure 1b). The SOM portraits of these regions exhibit a similar upregulation of genes in the upper left corner of the map, with subtle variations in MEI/SEB‐DUCT spots due to their admixture with acinar cells (see previous subsection). To facilitate detailed comparisons, we linked regions of coordinated gene upregulation within the SOM portraits using module co‐appearance maps (Figure 4a, lower part). The SEB map reveals module co‐expression patterns resembling the three MEI stages, thus suggesting similar functions. Furthermore, SOM analysis of IC‐2 inflammatory spots suggests overlapping cell types and functions between SEB, MEI, and IC‐2 spots based on shared expression patterns (Figure 4a, lower part).

For a deeper comparison, we analyzed 500 differentially expressed genes (DEGs) extracted from pseudotime (PT) analysis of MEI maturation (Figure 3d) and compared them with 500 DEGs derived from a similar PT analysis of SEB maturation in skin sebaceous gland hyperplasia (SGH) (Schmidt, Hansmann, et al., 2024). SGH, a common alteration of sebaceous glands in the elder, represents glands enlarged in size and number, but with otherwise normal cellular differentiation (Eisen & Michael, 2009; Iacobelli et al., 2017). More than 50% of these DEGs overlapped (Figure 4b) but exhibited stage‐specific shifts, as depicted in the Sankey‐flow diagram (Figure 4b, lower part). For instance, genes associated with the MG basal layer (MEI‐B) predominantly map to the first stage of SG maturation (SEB‐1), while SEB‐B genes are underrepresented in the MEI‐B stage. Conversely, genes from the MEI‐DIFF and MEI‐DUCT clusters converge in SEB‐3 of the SGH. These observations align with the finding that the basal layer transcriptome of the MEI overlaps with IC‐2 spots (see SOM portraits in Figure 4a and the UMAP of the EL in Figure 4e,f, below), and MEI‐DUCT spots are shared with SEB‐DUCT spots in the EL image (Figure 4a). Despite these shifts, MG and skin sebaceous maturation match well.

We further performed DEG analysis between MEI, subsuming all genes from the stages MEI‐B and MEI‐DIFF, and EL‐SEB (Table S1), as well as from MEI and SGH‐SEB stages, to identify differences in transcriptional programs underlying meibum and sebum secretion in the EL and between the EL and skin SGH, respectively (Figure 4c). Notably, genes such as *ALOX15B* and *DGAT2L6* (sebaceous glands), or *AQP9*, *MMP3*, and *PITX1* (MG) pop up in both comparisons as shared genes. A significant overlap was observed in the DEGs upregulated and downregulated in these comparisons, respectively (Figure 4e). The smaller number of upregulated genes in EL‐SEB is likely due to the limited number of related spots.

Next, we explored the biological context of these DEGs, identifying signatures related to “mitochondrion” and “oxidative phosphorylation” as well as “lipid metabolism” and “peroxisome” overexpressed in SEB, while signatures such as “endoplasmic reticulum,” “antigen binding,” “protein kinase,” and “SH3 domain” were differentially activated in MEI (Figure 4d). To comprehensively compare the meibocyte and sebocyte transcriptomes in the EL and SGH images, we integrated the UMAPs of their respective spots (Figure 4e). Relevant spots aggregated in a lower‐left branch/cluster in the UMAP. Separate UMAP plots for EL and SG revealed intriguing differences (Figure 4f): SEB‐B and SEB‐1 form a separate cluster, distinct from MEI‐B, which is consistent with the observed stage‐specific shifts between SEB and MEI maturation. Notably, IC‐2 spots from the EL image cluster near basal layer spots (MEI‐B and SEB‐B), suggesting a regulatory role in meibum and sebum production on the basal layer.

### 3.6 Immunofluorescence confirms expression of proteins selectively expressed in MGs or sebaceous glands

**FIGURE 5:**
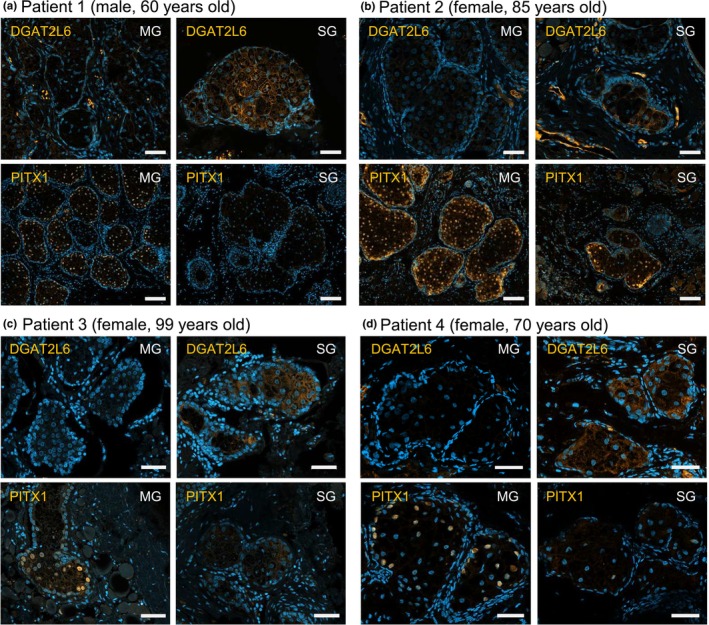
Expression of PITX1 and DGAT2L6 in MG compared to sebaceous glands. Immunofluorescent staining of DGAT2L6 and PITX1 (both labeled with Cy3 in orange) in meibomian glands (MG) and sebaceous glands (SG) of 4 different patients (age and gender are indicated; the sample employed for the spatial transcriptome analysis was obtained from patient 1). The nuclei are stained with DAPI (blue). Scale bars represent 100 μm in the lower line of A and B and 50 μm elsewhere. In general, DGAT2L6 expression was stronger in SGs compared to MGs. In contrast, PITX1 was mainly found in the nuclei of cells within the MGs.

To confirm that the transcripts detected as typical for MG compared to SG (and vice versa) are translated in this way, we detected PITX1 and DGAT2L6 (see Figure 4c) by immunofluorescence in the eyelid sample employed for ST analysis (patient 1). PITX1, a transcription factor known to regulate hindlimb tissue patterning and pituitary development (Tran & Kioussi, 2021), shows widespread expression in the nuclei of MG cells but is absent in eyelid SG cells (Figure 5a). DGAT2L6, a key enzyme of the sebum synthesis pathway (Lopes‐Marques et al., 2019), shows strong expression in SG compared to weak expression in MG cells (Figure 5a). To demonstrate that such an expression pattern is not a singularity of the employed sample, we surveyed additional eyelid samples and identified three further patients whose samples contained both MG and SGs (patients 2–4). While less clear for patient 2, the staining of patients 3 and 4 confirmed the expression pattern observed in patient 1, with stronger expression of PITX1 in the MG compared to the SG, and the opposite pattern for DGAT2L6 (Figure 5b).

## 4 DISCUSSION

The transcriptome data reported here from patient 1 provide an initial portrait for investigating MG function in normal and pathological contexts. The ST analysis of the human eyelid (1) provided a catalog of cell types in the eyelid, including the epidermis, the conjunctiva, MG, and sebaceous glands; (2) revealed the set of transcriptional changes taking place as human meibocytes differentiate and produce meibum, and (3) allowed assessing similarities and differences in the transcriptional program of MGs and sebaceous glands, in part confirmed by assessing an independent data set and an additional eyelid samples.

### 4.1 Cell types identified in the eyelid

With an unsupervised approach based on gene expression similarities combined with histological assessment, we identified 18 cell states, including three clusters comprising human meibomian gland cells (MEI‐B, MEI‐DIFF, and MEI‐DUCT). In comparison, scRNAseq of mouse tarsal plates allowed the identification of 14 clusters representing different cell types, including four clusters putatively corresponding to meibocytes at different stages of differentiation/maturation (Butovich & Wilkerson, 2022). Another scRNAseq‐based study of the MG cells resulted in the formation of seven clusters, three forming the lipid‐producing acinar cells and four constituting the ductal epithelium (Wiedemann et al., 2024). Wiedemann and colleagues also reported the spatial analysis of MG transcriptome using a set of 100 probes, which resulted in 13 cell states, including three MG clusters (Wiedemann et al., 2024). Finally, Zhu and colleagues (Zhu et al., 2025) recently reported untargeted, genome‐wide ST analysis with the 10× Visium Spatial Gene Expression platform. The ST data revealed that the authors' annotation of MG cell populations based on single‐nucleus RNA sequencing (snRNA‐seq) was congruent with their anatomical positions within the tarsal plate, but the spatial data was not reported in detail so far.

SOM‐portrayal of ST images and pseudotime analysis confirmed progress from MEI‐B to MEI‐DIFF (and to MEI‐DUCT, also including MG and sebaceous gland duct cells), reflecting the differentiation program that leads to meibum production. Accordingly, gene set enrichment analysis associated meibocyte maturation with energy and lipid metabolism in early and intermediate stages (MG acinar cells) and later with barrier and epithelial functions (ductal cells). The co‐clustering of the meibomian and sebaceous ducts indicates so far unappreciated similarities of these ductal structures.

### 4.2 The meibomian and sebaceous gland transcriptome display both similar and unique features

While MG and sebaceous glands were included in the module *lipid metabolism*, the UMAP of the spots separates cell type‐specific clusters of MGs from sebaceous glands, which is in concordance with the different gland morphology and dissimilar composition of meibum compared to sebum (Liu et al., 2024). The MG‐specific transcriptional signature included the terms *endoplasmic reticulum*, *antigen binding*, *protein kinase*, and *SH3 domain*, which were brought about by transcripts including *AQP9*, *FABP5*, *ELOVL6*, *MMP3*, *and PITX1*. Expression of genes such as *FABP5* (Qi et al., 2024) and *AQP9* (Yu et al., 2012) has been previously reported in the MG and is most likely associated with the import of substrates for meibum production. Another “MG gene”, the endoplasmic reticulum enzyme *ELOVL6* (Matsuzaka, 2021), catalyzes chain elongation of C12–C16 saturated and monounsaturated fatty acids to form C18 fatty acids such as stearate (C18:0) and oleate (C18:1n‐9) and seems therefore important for the meibum‐specific lipid composition. MMP3 belongs to a family of extracellular zinc‐dependent endopeptidases (Page‐McCaw et al., 2007), which contribute to various physiological and pathological processes including cell invasion and metastasis, the regulation of multiple signaling pathways and immunological functions. Interestingly, *MMP3* was recently shown to be an appealing target for treating glaucoma (O'Callaghan et al., 2023). Notably, we confirmed MG‐specific expression of PITX1, a transcription factor with known functions in hindlimb tissue patterning and pituitary development (Tran & Kioussi, 2021), at the protein level and in additional eyelid samples. As mice lacking PITX1 die during embryonic development or shortly after birth due to severe hindlimb defects (Lanctôt et al., 1999; Szeto et al., 1999), addressing the role of this transcription factor in MG development and homeostasis will require a tissue‐specific gene targeting model.

### 4.3 Limitations of the study

The 10× Visium technology employed for ST imaging of the MG features a spot diameter of 55 μm, corresponding to approximately seven to 12 cells per spot. This resolution clearly does not match single‐cell resolution and does therefore not allow for the precise imaging of layered structures with an estimated thickness of only one to two cell layers, such as the basal layers or the ductal regions of the MG or sebaceous gland. This limitation may explain some discrepancies between our data and previous studies. For example, *AWAT2* has been identified predominantly in differentiated meibocytes in humans and mice (Rho et al., 2022; Wiedemann et al., 2024), while it was detected at high levels in both basal and differentiated meibocytes in our study. Thus, the precise delineation between successive differentiation stages of meibocyte maturation remains somewhat uncertain. Nevertheless, pseudotime analysis and the identification of submodules provide an adequate depiction of ST imaging by capturing shifts in transcriptional programs. Furthermore, the classification of meibocyte maturation into stages represents a conceptual model designed to better describe changes along the differentiation trajectory, including the activation of key genes. In reality, this process is likely more continuous, particularly between the MEI‐B and MEI‐DIFF stages, without distinct transition boundaries. Also, most of the MGs in the ST image are sectioned at different angles. This variability introduces geometric distortions that complicate the precise assignment of spots to different classes. The self‐organizing map (SOM) portrayal method enables the deconvolution of transcriptional programs within each spot into modules corresponding to distinct cell types. Thus, the co‐occurrence of expression modules A and C in the MEI‐DUCT spots indicates a combination of mature meibocytes and ductal epithelial cells.

The recently developed 10× Visium HD technology offers a substantially improved spatial resolution, with a spot size of approximately 2 μm, approaching single‐cell resolution. However, due to the sparsity of sequencing reads, the recommended approach is to aggregate spots into larger bins of 8–16 μm. While 10× Visium HD was not available for this study—which, to our knowledge, represents the first ST analysis of the human MG—this technology is expected to enhance spatial resolution, albeit likely falling short of its nominal resolution.

Accordingly, a recent single‐cell (non‐spatial) study of the mouse eyelid (Zhu et al., 2025) did identify more cell‐type clusters compared to our ST spot clusters (32 versus 18 in total), with a similar ratio for the MG (7 vs. 4, considering IC2 as mixed with MEI‐B). The higher granularity observed in most scRNA‐seq clusters is primarily attributable to differences in cell states rather than distinct cell types. A single cell‐resolved ST analysis will represent an important future milestone in eyelid transcriptomics.

Another limitation of this study is the absence of biological replicates for the eyelid (EL) spatial transcriptomics (ST) analysis. However, it is important to note that our ST image includes multiple MG acini, which can be considered intrasample replicates (or “technical replicates”) that capture a significant degree of spatial variability, including meibocytes at various differentiation stages along the maturation trajectory. Particularly, the different maturation stages are consistently observed in different MGs across the eyelid (Figure 1c and Figure 3b,c). Importantly, we confirmed the expression of *PITX1* and *DGAT2L6* as meibomian‐ and sebaceous gland‐selective transcripts, respectively, by detecting the corresponding proteins in additional, independent eyelid samples. Finally, this study did not address pathological or age‐related changes in the EL transcriptome.

## 5 CONCLUSIONS

In summary, our report integrates spatial transcriptomics, pseudotime analysis, and functional enrichment to deliver a high resolution and comprehensive portrait of the transcriptional dynamics underlying meibocyte differentiation. The data support continuous meibocyte development by consecutive modulation of cellular functions that can be associated with specific gene sets and display striking differences compared to sebocyte differentiation. Regarding the later point, we provide the first systematic comparison between the meibomian and sebaceous transcriptional programs, detecting known and novel transcripts expressed (almost) exclusively in each gland type. Our data also delineate future experimental approaches of translational relevance, as it delivers novel candidates to modulate meibocyte function in MG diseases.

## AUTHOR CONTRIBUTIONS

H.B. and M.R.S. conceived and designed research, interpreted results of experiments, prepared figures, drafted, edited, and revised the manuscript. U.H., H.L.‐W., F.H., and H.P. performed experiments and approved the final version of the manuscript. M.S. analyzed data, performed experiments, and approved the final version of the manuscript.

## FUNDING INFORMATION

H.B. acknowledges the support of the Higher Education and Science Committee of the Republic of Armenia in the frame of the project 25RL‐1F024F005.

## CONFLICT OF INTEREST STATEMENT

The authors declare that they have no competing interests.

## ETHICS STATEMENT

The eyelid samples employed in this study were collected at the Department of Ophthalmology of the University of Leipzig during lateral tarsal strip procedure for ectropion correction and after patient consent for use of the specimen for research purposes, and in full compliance with the Declaration of Helsinki principles.

## Supporting information


Figure S1.

Table S1.

Table S2.

## Data Availability

Datasets related to this article can be found at the public Leipzig Health Atlas repository (https://www.health‐atlas.de/) under the accession number LHA ID: 8MWXC02K01‐6. Together with this publication, we provide an interactive web browser for inspecting the ST data of the eyelid (http://gondwanaland.izbi.uni‐leipzig.de:5978/?dataset=17). The interactive web browser also provides the SGH data under the link http://gondwanaland.izbi.uni‐leipzig.de:5978/?dataset=16.

## References

[phy270571-bib-0001] Butovich, I. A. (2013). Tear film lipids. Experimental Eye Research, 117, 4–27. 10.1016/j.exer.2013.05.010 23769846 PMC3844095

[phy270571-bib-0002] Butovich, I. A. (2017). Meibomian glands, meibum, and meibogenesis. Experimental Eye Research, 163, 2–16. 10.1016/j.exer.2017.06.020 28669846 PMC5728685

[phy270571-bib-0003] Butovich, I. A. , Bhat, N. , & Wojtowicz, J. C. (2019). Comparative transcriptomic and lipidomic analyses of human male and female Meibomian glands reveal common signature genes of meibogenesis. International Journal of Molecular Sciences, 20, 4539. 10.3390/ijms20184539 31540257 PMC6769918

[phy270571-bib-0004] Butovich, I. A. , Lu, H. , McMahon, A. , & Eule, J. C. (2012). Toward an animal model of the human tear film: Biochemical comparison of the mouse, canine, rabbit, and human meibomian lipidomes. Investigative Ophthalmology & Visual Science, 53, 6881–6896. 22918629 10.1167/iovs.12-10516 PMC3466071

[phy270571-bib-0005] Butovich, I. A. , & Suzuki, T. (2020). Delineating a novel metabolic high triglycerides‐low waxes syndrome that affects lipid homeostasis in meibomian and sebaceous glands. Experimental Eye Research, 199, 108189. 10.1016/j.exer.2020.108189 32805264 PMC7592343

[phy270571-bib-0006] Butovich, I. A. , & Wilkerson, A. (2022). Dynamic changes in the gene expression patterns and lipid profiles in the developing and maturing Meibomian glands. International Journal of Molecular Sciences, 23, 7884. 10.3390/ijms23147884 35887230 PMC9321132

[phy270571-bib-0007] Butovich, I. A. , Wilkerson, A. , Bhat, N. , McMahon, A. , & Yuksel, S. (2019). On the pivotal role of Elovl3/ELOVL3 in meibogenesis and ocular physiology of mice. The FASEB Journal, 33, 10034–10048. 10.1096/fj.201900725R 31208226 PMC6704448

[phy270571-bib-0008] Butovich, I. A. , Wilkerson, A. , & Yuksel, S. (2021). Depletion of cholesteryl esters causes meibomian gland dysfunction‐like symptoms in a Soat1‐null mouse model. International Journal of Molecular Sciences, 22, 1583. 10.3390/ijms22041583 33557318 PMC7915537

[phy270571-bib-0009] Cao, J. , Spielmann, M. , Qiu, X. , Huang, X. , Ibrahim, D. M. , Hill, A. J. , Zhang, F. , Mundlos, S. , Christiansen, L. , Steemers, F. J. , Trapnell, C. , & Shendure, J. (2019). The single‐cell transcriptional landscape of mammalian organogenesis. Nature, 566, 496–502. 10.1038/s41586-019-0969-x 30787437 PMC6434952

[phy270571-bib-0010] Dahlhoff, M. , Camera, E. , Schafer, M. , Emrich, D. , Riethmacher, D. , Foster, A. , Paus, R. , & Schneider, M. R. (2016). Sebaceous lipids are essential for water repulsion, protection against UVB‐induced apoptosis and ocular integrity in mice. Development (Cambridge, England), 143, 1823–1831. 10.1242/dev.132753 26989175

[phy270571-bib-0011] de Souza, G. A. , Godoy, L. M. F. , & Mann, M. (2006). Identification of 491 proteins in the tear fluid proteome reveals a large number of proteases and protease inhibitors. Genome Biology, 7, R72. 10.1186/gb-2006-7-8-R72 16901338 PMC1779605

[phy270571-bib-0012] Dilly, P. N. (1994). Structure and function of the tear film. Advances in Experimental Medicine and Biology, 350, 239–247. 10.1007/978-1-4615-2417-5_41 8030483

[phy270571-bib-0013] Ehrmann, C. , & Schneider, M. R. (2016). Genetically modified laboratory mice with sebaceous glands abnormalities. Cellular and Molecular Life Sciences, 73, 4623–4642. 10.1007/s00018-016-2312-0 27457558 PMC11108334

[phy270571-bib-0014] Eisen, D. B. , & Michael, D. J. (2009). Sebaceous lesions and their associated syndromes: Part I. Journal of the American Academy of Dermatology, 61, 549–560. 19751879 10.1016/j.jaad.2009.04.058

[phy270571-bib-0015] Franck, C. (1991). Fatty layer of the precorneal film in the ‘office eye syndrome’. Acta Ophthalmologica, 69, 737–743. 10.1111/j.1755-3768.1991.tb02052.x 1789088

[phy270571-bib-0053] Hao, Y. , Stuart, T. , Kowalski, M. H. , Choudhary, S. , Hoffman, P. , Hartman, A. , Srivastava, A. , Molla, G. , Madad, S. , Fernandez‐Granda, C. , & Satija, R. (2024). Dictionary learning for integrative, multimodal and scalable single‐cell analysis. Nature Biotechnology, 42, 293–304. 10.1038/s41587-023-01767-y PMC10928517 37231261

[phy270571-bib-0016] Iacobelli, J. , Harvey, N. T. , & Wood, B. A. (2017). Sebaceous lesions of the skin. Pathology, 49, 688–697. 10.1016/j.pathol.2017.08.012 29078997

[phy270571-bib-0017] Knop, E. , Knop, N. , Millar, T. , Obata, H. , & Sullivan, D. A. (2011). The international workshop on meibomian gland dysfunction: Report of the subcommittee on anatomy, physiology, and pathophysiology of the meibomian gland. Investigative Ophthalmology & Visual Science, 52, 1938–1978. 21450915 10.1167/iovs.10-6997c PMC3072159

[phy270571-bib-0018] Kumar, A. , Kumar Dorairaj, S. , Prabhakaran, V. C. , Prakash, D. R. , & Chakraborty, S. (2007). Identification of genes associated with tumorigenesis of meibomian cell carcinoma by microarray analysis. Genomics, 90, 559–566. 10.1016/j.ygeno.2007.07.008 17889501

[phy270571-bib-0019] Lanctôt, C. , Moreau, A. , Chamberland, M. , Tremblay, M. L. , & Drouin, J. (1999). Hindlimb patterning and mandible development require the Ptx1 gene. Development, 126, 1805–1810. 10.1242/dev.126.9.1805 10101115

[phy270571-bib-0020] Liu, Y. , Butovich, I. A. , Garreis, F. , Zahn, I. , Scholz, M. , Gaffling, S. , Jabari, S. , Dietrich, J. , & Paulsen, F. (2024). Comparative characterization of human meibomian glands, free sebaceous glands, and hair‐associated sebaceous glands based on biomarkers, analysis of secretion composition, and gland morphology. International Journal of Molecular Sciences, 25, 3109. 10.3390/ijms25063109 38542083 PMC10970278

[phy270571-bib-0021] Löffler‐Wirth, H. , Kalcher, M. , & Binder, H. (2015). oposSOM: R‐package for high‐dimensional portraying of genome‐wide expression landscapes on bioconductor. Bioinformatics, 31, 3225–3227. 10.1093/bioinformatics/btv342 26063839

[phy270571-bib-0022] Lopes‐Marques, M. , Machado, A. M. , Alves, L. Q. , Fonseca, M. M. , Barbosa, S. , Sinding, M.‐H. S. , Rasmussen, M. H. , Iversen, M. R. , Frost Bertelsen, M. , Campos, P. F. , da Fonseca, R. , Ruivo, R. , & Castro, L. F. C. (2019). Complete inactivation of sebum‐producing genes parallels the loss of sebaceous glands in Cetacea. Molecular Biology and Evolution, 36, 1270–1280. 10.1093/molbev/msz068 30895322 PMC6526905

[phy270571-bib-0023] Matsuzaka, T. (2021). Role of fatty acid elongase Elovl6 in the regulation of energy metabolism and pathophysiological significance in diabetes. Diabetology International, 12, 68–73. 10.1007/s13340-020-00481-3 33479581 PMC7790921

[phy270571-bib-0024] McMahon, A. , Lu, H. , & Butovich, I. A. (2014). A role for ELOVL4 in the mouse meibomian gland and sebocyte cell biology. Investigative Ophthalmology & Visual Science, 55, 2832–2840. 24677106 10.1167/iovs.13-13335 PMC4008046

[phy270571-bib-0025] Millar, T. J. , & Schuett, B. S. (2015). The real reason for having a meibomian lipid layer covering the outer surface of the tear film—A review. Experimental Eye Research, 137, 125–138. 10.1016/j.exer.2015.05.002 25981748

[phy270571-bib-0026] Miyazaki, M. , Man, W. C. , & Ntambi, J. M. (2001). Targeted disruption of stearoyl‐CoA desaturase1 gene in mice causes atrophy of sebaceous and meibomian glands and depletion of wax esters in the eyelid. The Journal of Nutrition, 131, 2260–2268. 11533264 10.1093/jn/131.9.2260

[phy270571-bib-0027] O'Callaghan, J. , Delaney, C. , O'Connor, M. , van Batenburg‐Sherwood, J. , Schicht, M. , Lütjen‐Drecoll, E. , Hudson, N. , Ni Dhubhghaill, S. , Humphries, P. , Stanley, C. , Keravala, A. , Chalberg, T. , Lawrence, M. S. , & Campbell, M. (2023). Matrix metalloproteinase‐3 (MMP‐3)‐mediated gene therapy for glaucoma. Science Advances, 9, eadf6537. 10.1126/sciadv.adf6537 37075118 PMC10115410

[phy270571-bib-0028] Page‐McCaw, A. , Ewald, A. J. , & Werb, Z. (2007). Matrix metalloproteinases and the regulation of tissue remodelling. Nature Reviews. Molecular Cell Biology, 8, 221–233. 10.1038/nrm2125 17318226 PMC2760082

[phy270571-bib-0029] Parfitt, G. J. , Brown, D. J. , & Jester, J. V. (2016). Transcriptome analysis of aging mouse meibomian glands. Molecular Vision, 22, 518–527. 27279727 PMC4880544

[phy270571-bib-0030] Parfitt, G. J. , Xie, Y. , Geyfman, M. , Brown, D. J. , & Jester, J. V. (2013). Absence of ductal hyper‐keratinization in mouse age‐related meibomian gland dysfunction (ARMGD). Aging, 5, 825–834. 10.18632/aging.100615 24259272 PMC3868725

[phy270571-bib-0031] Qi, X. , Yang, Y. , Xiong, D. , Wu, S. , Cui, G. , & Zhang, Q. (2024). ER‐1 deficiency induces inflammation and lipid deposition in meibomian gland and lacrimal gland. Biochemical and Biophysical Research Communications, 696, 149526. 10.1016/j.bbrc.2024.149526 38241812

[phy270571-bib-0032] Rao, A. , Barkley, D. , França, G. S. , & Yanai, I. (2021). Exploring tissue architecture using spatial transcriptomics. Nature, 596, 211–220. 10.1038/s41586-021-03634-9 34381231 PMC8475179

[phy270571-bib-0033] Rho, C. R. , Kim, S. W. , Lane, S. , Gao, F. , Kim, J. , Xie, Y. , Brown, D. J. , Skowronska‐Krawczyk, D. , & Jester, J. V. (2022). Expression of acyl‐CoA wax‐alcohol acyltransferase 2 (AWAT2) by human and rabbit meibomian glands and meibocytes. The Ocular Surface, 23, 60–70. 10.1016/j.jtos.2021.11.010 34838721 PMC10393063

[phy270571-bib-0034] Sawai, M. , Watanabe, K. , Tanaka, K. , Kinoshita, W. , Otsuka, K. , Miyamoto, M. , Sassa, T. , & Kihara, A. (2021). Diverse meibum lipids produced by Awat1 and Awat2 are important for stabilizing tear film and protecting the ocular surface. iScience, 24, 102478. 10.1016/j.isci.2021.102478 34113821 PMC8169949

[phy270571-bib-0035] Schmidt, M. , Avagyan, S. , Reiche, K. , Binder, H. , & Loeffler‐Wirth, H. (2024). A spatial transcriptomics browser for discovering gene expression landscapes across microscopic tissue sections. Current Issues in Molecular Biology, 46, 4701–4720. 10.3390/cimb46050284 38785552 PMC11119626

[phy270571-bib-0036] Schmidt, M. , Binder, H. , & Schneider, M. R. (2025). The metabolic underpinnings of sebaceous lipogenesis. Communications Biology, 8, 670. 10.1038/s42003-025-08105-9 40289206 PMC12034822

[phy270571-bib-0037] Schmidt, M. , Hansmann, F. , Loeffler‐Wirth, H. , Zouboulis, C. C. , Binder, H. , & Schneider, M. R. (2024). A spatial portrait of the human sebaceous gland transcriptional program. Journal of Biological Chemistry, 300, 107442. 10.1016/j.jbc.2024.107442 38838779 PMC11261126

[phy270571-bib-0038] Schneider, M. R. , & Paus, R. (2010). Sebocytes, multifaceted epithelial cells: Lipid production and holocrine secretion. The International Journal of Biochemistry & Cell Biology, 42, 181–185. 19944183 10.1016/j.biocel.2009.11.017

[phy270571-bib-0039] Szeto, D. P. , Rodriguez‐Esteban, C. , Ryan, A. K. , O'Connell, S. M. , Liu, F. , Kioussi, C. , Gleiberman, A. S. , Izpisúa‐Belmonte, J. C. , & Rosenfeld, M. G. (1999). Role of the Bicoid‐related homeodomain factor Pitx1 in specifying hindlimb morphogenesis and pituitary development. Genes & Development, 13, 484–494. 10.1101/gad.13.4.484 10049363 PMC316471

[phy270571-bib-0040] Tran, T. Q. , & Kioussi, C. (2021). Pitx genes in development and disease. Cellular and Molecular Life Sciences, 78, 4921–4938. 10.1007/s00018-021-03833-7 33844046 PMC11073205

[phy270571-bib-0041] Widjaja‐Adhi, M. A. K. , Silvaroli, J. A. , Chelstowska, S. , Trischman, T. , Bederman, I. , Sayegh, R. , & Golczak, M. (2020). Deficiency in acyl‐CoA:Wax alcohol acyltransferase 2 causes evaporative dry eye disease by abolishing biosynthesis of wax esters. The FASEB Journal, 34, 13792–13808. 10.1096/fj.202001191R 32851726 PMC7722226

[phy270571-bib-0042] Wiedemann, J. , Kashgari, G. , Lane, S. , Leonard, B. C. , Knickelbein, K. E. , Andersen, B. , & Jester, J. V. (2024). The effects of age and dysfunction on meibomian gland population dynamics. The Ocular Surface, 34, 194–209. 10.1016/j.jtos.2024.08.005 39122180 PMC11884644

[phy270571-bib-0043] Willcox, M. D. P. , Argüeso, P. , Georgiev, G. A. , Holopainen, J. M. , Laurie, G. W. , Millar, T. J. , Papas, E. B. , Rolland, J. P. , Schmidt, T. A. , Stahl, U. , Suarez, T. , Subbaraman, L. N. , Uçakhan, O. Ö. , & Jones, L. (2017). TFOS DEWS II tear film report. The Ocular Surface, 15, 366–403. 10.1016/j.jtos.2017.03.006 28736338 PMC6035753

[phy270571-bib-0044] Wirth, H. , Löffler, M. , von Bergen, M. , & Binder, H. (2011). Expression cartography of human tissues using self organizing maps. BMC Bioinformatics, 12, 306. 10.1186/1471-2105-12-306 21794127 PMC3161046

[phy270571-bib-0045] Yaba, A. , Thalheim, T. , & Schneider, M. R. (2024). The role of cell‐cell and cell‐matrix junctional complexes in sebaceous gland homeostasis and differentiation. Cell Communication and Signaling, 22, 445. 10.1186/s12964-024-01835-z 39313816 PMC11421122

[phy270571-bib-0046] Yang, X. , Reneker, L. W. , Zhong, X. , Huang, A. J. W. , & Jester, J. V. (2023). Meibomian gland stem/progenitor cells: The hunt for gland renewal. The Ocular Surface, 29, 497–507. 10.1016/j.jtos.2023.07.004 37422152 PMC10528929

[phy270571-bib-0047] Yang, X. , Zhong, X. , Lin, H. , Huang, A. J. W. , & Reneker, L. W. (2024). Deletion of Fgfr2 in ductal basal epithelium with tamoxifen induces obstructive Meibomian gland dysfunction. Investigative Ophthalmology & Visual Science, 65, 36. 10.1167/iovs.65.13.36 PMC11578149 39546290

[phy270571-bib-0048] Yazdani, M. , Elgstøen, K. B. P. , Rootwelt, H. , Shahdadfar, A. , Utheim, Ø. A. , & Utheim, T. P. (2019). Tear metabolomics in dry eye disease: A review. International Journal of Molecular Sciences, 20, 3755. 10.3390/ijms20153755 31374809 PMC6695908

[phy270571-bib-0049] Yu, D. , Thelin, W. R. , Randell, S. H. , & Boucher, R. C. (2012). Expression profiles of aquaporins in rat conjunctiva, cornea, lacrimal gland and Meibomian gland. Experimental Eye Research, 103, 22–32. 10.1016/j.exer.2012.07.005 22828047

[phy270571-bib-0050] Zheng, Y. , Prouty, S. M. , Harmon, A. , Sundberg, J. P. , Stenn, K. S. , & Parimoo, S. (2001). Scd3‐‐a novel gene of the stearoyl‐CoA desaturase family with restricted expression in skin. Genomics, 71, 182–191. 11161812 10.1006/geno.2000.6429

[phy270571-bib-0051] Zhu, X. , Xu, M. , Portal, C. , Lin, Y. , Ferdinand, A. , Peng, T. , Morrisey, E. E. , Dlugosz, A. A. , Castellano, J. M. , Lee, V. , Seykora, J. T. , Wong, S. Y. , Iomini, C. , & Millar, S. E. (2025). Identification of Meibomian gland stem cell populations and mechanisms of aging. Nature Communications, 16, 1663. 10.1038/s41467-025-56907-6 PMC11830078 39955307

[phy270571-bib-0052] Zou, S. , Liu, J. , Si, H. , Huang, D. , Qi, D. , Pei, X. , Lu, D. , Huang, S. , & Li, Z. (2023). High‐fat intake reshapes the circadian transcriptome profile and metabolism in murine meibomian glands. Frontiers in Nutrition, 10, 1146916. 10.3389/fnut.2023.1146916 37006922 PMC10062204

