# Supplementary material for: Spatial transcriptome analysis of the human eyelid depicts meibomian gland cell differentiation: A pilot study
Source: Physiol Rep. 2025 Sep 19;13(18):e70571. doi: 10.14814/phy2.70571 (PMC12446996; doi:10.14814/phy2.70571)
Supplement: Supplementary file 1 — Figure S1. [file PHY2-13-e70571-s001.pdf]

Supplemental Figure S1

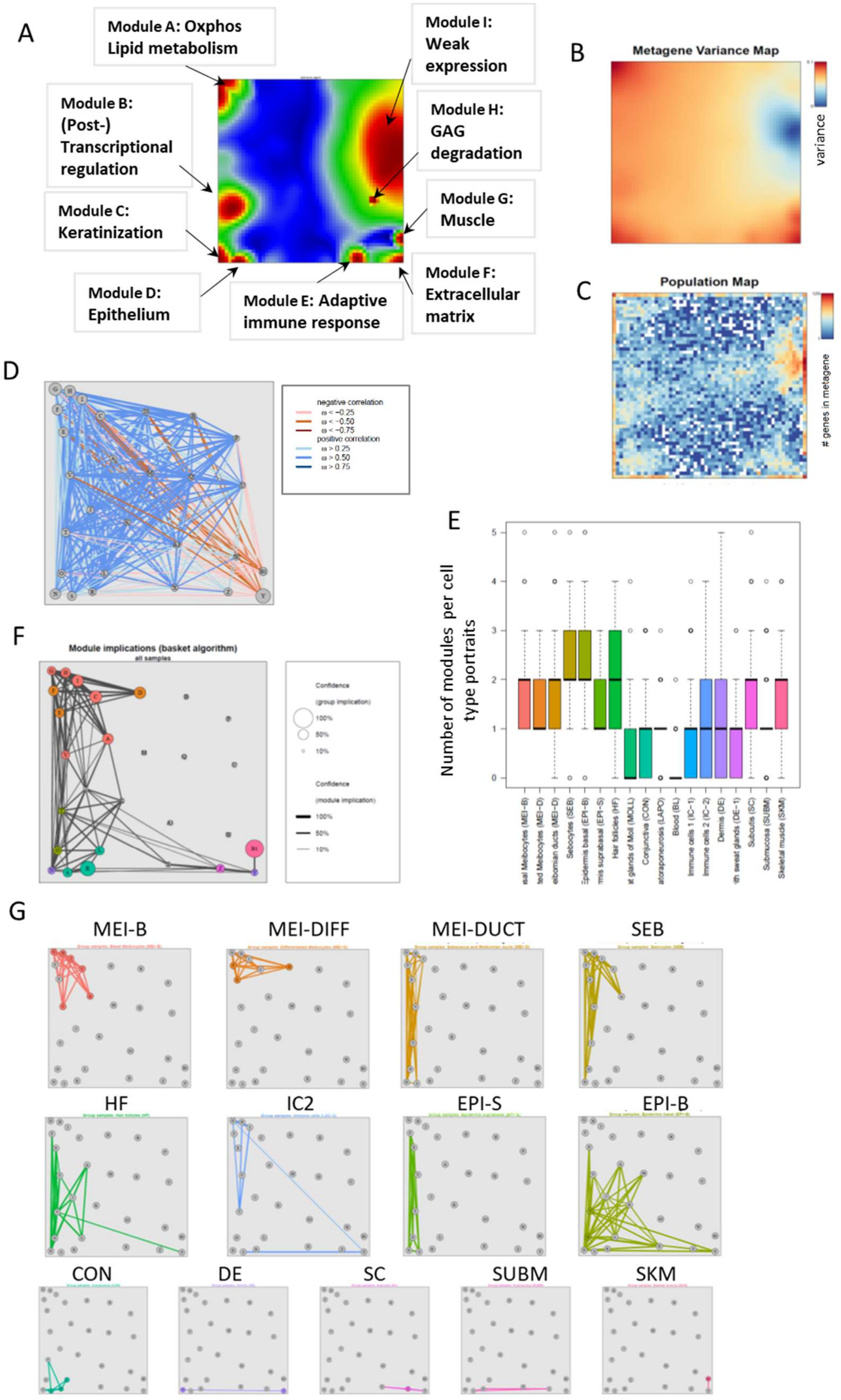

Figure S1: Topology of the Transcriptomic Landscape of the Human Eyelid

(A) The summary module map provides an overview of the modules of co-regulated genes, highlighting their upregulation in specific cell types and associated functions.

(B) The variance map reveals that most cell type-associated modules exhibit high expression variance (dark brown), whereas the "blood" module (Module I) contains genes with minimal variance.

(C) The population map depicts the density of genes in each pixel, showing that genes tend to cluster within or near modules, including the invariant genes in Module I.

(D) The correlation map represents relationships between modules: positively correlated modules are connected by blue lines, while negatively correlated modules are linked by red lines. Modules on the left side of the map (associated with gland maturation and epidermal cells) exhibit strong mutual co-expression and are generally anti-correlated with modules in the lower-right region (linked to muscle, stroma, and inflammatory cells), indicating opposing transcriptional programs.

(E) The boxplot illustrates the number of co-expressed modules in individual self-organizing map (SOM) portraits for each cell type. The vertical line within each box denotes the mean number of modules. On average, gland- and epidermis-related cell types co-express one to two modules, whereas muscle, stroma, and inflammatory cell types typically express a single module.

(F) The map of mutual module expression connects modules that are jointly expressed across individual portraits, revealing prominent co-expression patterns within the modules associated with gland and epidermal cell types on the left side of the map while modules at the right side appear mostly alone.

(G) The mutual module expression map, shown separately for each spot type, highlights distinct patterns of co-expression across different modules. The analysis reveals that spots corresponding to stages of meibocyte differentiation exhibit overlapping module expression centered around Module A, with a systematic shift toward epidermal spots in Mei-DUCT. Sebocyte (SEB)-related spots show a co-expression pattern that aligns broadly with that of Mei-B, Mei-DIFF, and Mei-DUCT, encompassing the entire meibocyte maturation program and indicating close similarities between these gland types. IC2-related spots display a clear mix of meibocyte and inflammatory characteristics, whereas HF-spots exhibit slightly distinct module activation patterns, particularly around Module B. Epidermis-related spots (EPI-S, EPI-B, CON) predominantly activate modules located in the lower-left corner of the map, while skeletal muscle and inflammatory spots (SKM, IC1) are marked by distinct module co-activation patterns in the lower-right part of the map.
